# Association Between Parkinson’s Disease and Melanoma: Putting the Pieces Together

**DOI:** 10.3389/fnagi.2020.00060

**Published:** 2020-03-10

**Authors:** Qing Ye, Ya Wen, Nasser Al-Kuwari, Xiqun Chen

**Affiliations:** ^1^Department of Neurology, Massachusetts General Hospital, Harvard Medical School, Boston, MA, United States; ^2^Ietheory Institute, Burlington, MA, United States

**Keywords:** Parkinson’s disease, melanoma, cancer, risk factors, pigmentation

## Abstract

Patients with Parkinson’s disease (PD) generally have reduced risk of developing many types of cancers, except melanoma—a malignant tumor of melanin-producing cells in the skin. For decades, a large number of epidemiological studies have reported that the occurrence of melanoma is higher than expected among subjects with PD, and the occurrence of PD is reciprocally higher than expected among patients with melanoma. More recent epidemiological studies further indicated a bidirectional association, not only in the patients themselves but also in their relatives. This association between PD and melanoma offers a unique opportunity to understand PD. Here, we summarize epidemiological, clinical, and biological evidence in regard to shared risk factors and possible underlying mechanisms for these two seemingly distinct conditions.

## Introduction

Epidemiological studies have provided substantial insight into our understanding of Parkinson’s disease (PD). Many risks and inverse risk factors have been identified to play important roles in the development and progression of the disease (Ascherio and Schwarzschild, 2016). The association between PD and melanoma discovered by epidemiological studies appears to be particularly intriguing. Melanoma is a form of cancer that develops from melanocytes, which produce melanin, the pigment that colors skin, eyes, and hair. Melanoma is among the most aggressive and treatment-resistant human cancers and it accounts for 75% of all skin cancer deaths. PD and cancer are distinct diseases, with cancer resulting from uncontrolled cell growth and proliferation whereas PD is characterized by the premature death of dopaminergic neurons. Epidemiological evidence indeed supports an overall reduced risk of total cancer in patients with PD (Zhang and Liu, 2019), and reduced risk of developing PD in cancer patients (Bajaj et al., 2010; Fois et al., 2010; Tacik et al., 2016; Cui et al., 2019; Zhang and Liu, 2019). This is, however, not the case with melanoma. A large number of epidemiological studies have reported that the occurrence of melanoma was higher than expected among subjects with PD, and melanoma patients were reciprocally more likely to develop PD (Huang et al., 2015; Bose et al., 2018). A meta-analysis showed that the risk of developing melanoma in individuals with PD was 3.6-fold higher than expected, with a pooled odds ratio of 2.1 from 12 studies (Liu et al., 2011). A more recent meta-analysis reported a 2.4-fold increased risk of melanoma in PD and an overall pooled odds ratio of 1.83 from 22 studies (Huang et al., 2015).

## Shared Risk Factors and Inverse Risk Factors Between PD and Melanoma

Both PD and melanoma are considered multifaceted disorders that result from complex interactions of genetic and environmental factors. Growing evidence suggests that there are shared risk factors between PD and melanoma, which include:

**Age**. The risk of developing PD or melanoma increases with age. People typically develop PD around age 60 or older, while about half of melanomas occur in people over the age of 50.

**Family History and Genetics**. Having a close relative with PD increases the risk of developing PD, and having a close relative with melanoma also increases the risk of developing melanoma (Ward et al., 2017). The finding that a family history of melanoma in first-degree relatives was associated with a higher risk of PD suggests shared genetic predispositions (Gao et al., 2009a).

**Gender**. Men are 1.5 times more likely to develop PD (Wooten et al., 2004) or melanoma (National Cancer Institute, 2019; Rastrelli et al., 2014) than women.

**Ethnicity**. PD is more common in the white population than in black or Asian population (Van Den Eeden et al., 2003; Dahodwala et al., 2009; Wright Willis et al., 2010). Melanoma is also more common in the white population (Wooten et al., 2004).

**Red Hair and Fair Skin**. Melanoma is strongly linked to fair skin and red hair, the phenotype of the gene MC1R (melanocortin 1 receptor) polymorphisms. Red hair color has also been associated with an increased risk of developing PD (Chen et al., 2017b).

**Toxins**. Continuous exposure to environmental toxins such as pesticides has been reported to increase the risk of developing both PD (Goldman, 2014; Nandipati and Litvan, 2016) and melanoma (Dennis et al., 2010).

PD and melanoma also share inverse risk factors, such as:

**Smoking**. Many epidemiologic studies have consistently reported that PD was less common in people who smoke cigarette (Hernán et al., 2001, 2002; Ritz et al., 2007; Li et al., 2015), use smokeless tobacco (Benedetti et al., 2000; O’Reilly et al., 2005), and even only with environmental tobacco exposure (O’Reilly et al., 2009; Searles Nielsen et al., 2012). Smoking is usually associated with an increased risk of developing many types of cancer. Though surprisingly, studies have suggested that smokers had lower risks of melanoma than nonsmokers (Kessides et al., 2011; Dusingize et al., 2018). However, smoking was linked to an increased risk of melanoma metastases (Jones et al., 2017).

**Coffee Consumption**. Coffee consumption is associated with a decreased risk of developing PD, suggested by a large number of studies (Benedetti et al., 2000; Hernán et al., 2002; Hu et al., 2007; Costa et al., 2010). The risk of developing melanoma was also reported among different populations to be decreased in individuals who drink coffee (Loftfield et al., 2015; Wu et al., 2015; Lukic et al., 2016; Caini et al., 2017).

## Common Clinical Features of PD and Melanoma

Although their major symptoms are distinct, PD and melanoma share some common clinical features:

### Vitamin D Deficiency

Both PD and melanoma have lower serum vitamin D levels, which are associated with more severe symptoms. Lower serum vitamin D levels were found in patients with PD (Sleeman et al., 2017), and vitamin D deficiency was associated with increased risk of the disease (Sato et al., 1997; Evatt et al., 2008). Studies also found that in PD patients, vitamin D levels significantly correlated with falls and some non-motor symptoms such as insomnia, depression, and anxiety (Zhang et al., 2019), and lower serum 25-hydroxy vitamin D in early PD may predict greater disease severity (Sleeman et al., 2017). Conversely, higher plasma vitamin D levels were associated with lower symptom severity, better cognition, and less depression in PD patients without dementia (Peterson et al., 2013). Decreased serum vitamin level was also detected in patients with melanoma (Field and Newton-Bishop, 2011; Field et al., 2013), and lower levels of vitamin D were associated with thicker tumors and reduced patient survival (Slominski et al., 2017), tumor ulceration, and high tumor mitotic rate (Timerman et al., 2016; Moreno-Arrones et al., 2019).

### Sleep Disorders

Patients with PD often have sleep disorders (Menza et al., 2010). Sleep disturbance such as sleep apnea correlates with a higher risk of PD (Chen J.-C. et al., 2015; Yeh et al., 2016; Chou et al., 2017). The risk of melanoma was significantly higher in patients with sleep apnea as well (Gozal et al., 2016). Additionally, sleep apnea was reported to be associated with aggressive melanoma (Martínez-García et al., 2014; Martinez-Garcia et al., 2018).

### Extended Echogenic SN Area

Abnormally extended echogenic SN area was observed in both PD and melanoma patients by transcranial sonography (Rumpf et al., 2013). Later, a prospective observational study revealed that patients with melanoma had increased frequency of SN hyperechogenicity and prodromal motor and non-motor features of PD. This study also showed that larger echogenicity of SN correlated with lower serum iron in patients with melanoma, consistent with prior findings in PD (Walter et al., 2016).

## Possible Underlying Mechanisms for the PD and Melanoma Association

The epidemiological and clinical associations between PD and melanoma suggest common underlying mechanisms. Known PD- and melanoma-related genes/gene products and pathways have been implicated in the PD and melanoma associations.

## PD-Related Genes in Melanoma

**PRKN** gene encodes protein parkin, which belongs to a group of proteins called E3 ubiquitin ligases, and is involved in the maintenance of mitochondria, and also act as a tumor suppressor protein (Wahabi et al., 2018). More than 200 PRKN gene mutations were identified to cause PD, especially early-onset PD. PRKN mutations were also identified in melanoma cases, and its expression level was found to decrease in melanoma cell lines. PRKN somatic mutations were found in ~13% melanomas (Inzelberg et al., 2016b). Expression of PRKN in melanoma cells attenuated cell proliferation and conversely, inhibition of PRKN in melanocytes induced cell proliferation (Hu et al., 2016).

**SNCA** encodes protein alpha-synuclein (α-Syn), which is abundant in the brain. It plays a role in controlling the mobility of synaptic vesicles, regulating the release of neurotransmitters such as dopamine. α-Syn may interact with tyrosinase (TYR) and tyrosine hydroxylase (TH; Pan et al., 2012), which are enzymes involved in the biosynthesis of melanin and dopamine, respectively. Mutations in SNCA cause dementia with Lewy bodies, as well as PD. Elevated expression levels of α-Syn were indicated in skin cells from PD patients (Rodríguez-Leyva et al., 2014; Rodriguez-Leyva et al., 2017) and metastatic tissues from melanoma patients (Matsuo and Kamitani, 2010; Welinder et al., 2014). A study using melanoma cells showed that α-Syn might have inhibitory effects on melanin synthesis in melanoma cells (Pan et al., 2012). Some researchers proposed that neuromelanin might have direct interaction with α-Syn (Xu and Chan, 2015). However, whether α-Syn has a direct role in dermal melanin synthesis or melanoma is uncertain (Inzelberg et al., 2016a).

**LRRK2** encodes protein dardarin, which is involved in protein-protein interactions, kinase activity, and GTPase activity. LRRK2 mutations are the most common genetic cause of familial as well as sporadic PD. PD patients carrying LRRK2 G2019S mutation had a higher overall cancer risk (Agalliu et al., 2015) Although LRRK2 somatic mutations were demonstrated in human melanomas (Inzelberg et al., 2016b), melanoma is not among specific cancers that have been associated with LRRK2 mutations, which include leukemia, colon and skin cancer (Agalliu et al., 2019), breast cancer in women (Agalliu et al., 2015), and non-skin cancers (Inzelberg et al., 2012).

**PARK7** encodes protein DJ-1, with functions in regulating protein folding and oxidative stress. PARK7 mutations are associated with early-onset PD. DJ-1 is also considered as an oncogene (Vasseur et al., 2009). In melanoma cell lines, PARK7 expression is positively correlated with migratory ability (Cecconi et al., 2017), and patients with metastatic uveal melanoma had significantly higher levels of serum DJ-1 (Chen L.-L. et al., 2015).

**PINK1** encodes the protein PTEN induced kinase 1, a mitochondrial serine/threonine-protein kinase and a tumor suppressor. Mutations in this gene cause early-onset PD. PINK1 appears to protect mitochondria from stress-induced malfunctioning. PINK1 targets parkin to depolarized mitochondria to induce autophagy (Arena and Valente, 2017). Altered expression levels of PINK1 were detected in melanoma cells (Brown et al., 2017).

**PLA2G6** encodes the enzyme A2 phospholipase, which is involved in metabolizing phospholipids. Mutations in PLA2G6 cause early-onset parkinsonism (Tomiyama et al., 2011; Lin et al., 2018). PLA2G6 variants were associated with increased nevus counts, a major indicator for melanoma risk (Law et al., 2012).

**HTRA2** encodes a serine protease localized in the endoplasmic reticulum (ER) and mitochondria. It is regulated by PINK1 and is involved in apoptosis in PD (Strauss et al., 2005; Plun-Favreau et al., 2007; Desideri and Martins, 2012). It is reported that HTRA2 expression levels were changed in melanoma cells (Su et al., 2009).

**UCHL1** encodes a deubiquitinating enzyme called ubiquitin carboxy-terminal hydrolase L1, abundantly presents in all neurons. It is a PD susceptibility gene (Maraganore et al., 2004) and may link to alpha-synucleinopathy (Cartier et al., 2012). The aberrant expression level of UCHL1 was detected in melanoma cells (Wulfänger et al., 2013). It appeared to regulate melanin production and distribution in melanocytes (Seo et al., 2017).

## Cancer-or Melanoma-Related Genes in PD

**TRPM7** encodes protein Trpm7, which is an essential ion channel and serine/threonine-protein kinase. It regulates cellular calcium and magnesium levels. In melanocytes and melanoma cells, TRPM7 functions as a protector and detoxifier (Guo et al., 2012). TRPM7 channel was shown to regulate dopamine-dependent developmental transition and dopaminergic cell survival (Sun et al., 2015). Decreased TRPM7 expression was found in the SN area of PD patients (Sun et al., 2015).

**TP53** encodes p53 protein, a well-known tumor suppressor. It binds to DNA and triggers DNA damage repair or apoptosis. It is the most frequently mutated gene in human cancers and it plays broad roles in many types of cancers including melanoma. In PD, it may be responsible for inducing the death of dopaminergic neurons (Alves da Costa and Checler, 2011).

**PTEN** encodes protein Pten, which is also a major tumor suppressor. It involves in signaling pathways that regulate cell death. Similar to p53, PTEN is related to many cancers including melanoma (Aguissa-Touré and Li, 2012). *De novo* variants of PTEN were implicated in PD (Kun-Rodrigues et al., 2015), and PTEN was proposed to be a potential therapeutic target for the disease.

**GPNMB** encodes the protein transmembrane glycoprotein NMB, which is a transmembrane protein enriched on the cell surface of various cancer cells, including melanoma cells. It appears to have both inhibitory and tumor-promoter roles in different cancers (Taya and Hammes, 2018). Increased brain GPNMB expression may explain the association between the GPNMB gene locus and PD risk (Murthy et al., 2017).

**MC1R** encodes the melanocortin 1 receptor. In melanocytes, MC1R determines the relative amount of brown-black eumelanin and yellow-red pheomelanin by facilitating eumelanin production. Loss-of-function MC1R variants are associated with red hair, fair skin, poor tanning and increased risk of melanoma_._ Red hair color and loss-of-function MC1R R151C variant have also been associated with PD (Gao et al., 2009a; Chen et al., 2017b). Further, MC1R was reported to be expressed in dopaminergic neurons and it influenced dopaminergic neuron survival (Chen et al., 2017a).

## Other Genes That May Be Involved in Both PD and Melanoma

**CYP2D6** encodes the enzyme cytochrome P450 2D6, which catalyzes many reactions involved in drug metabolism. CYP2D6 polymorphisms have been associated with increased susceptibility to melanoma (Strange et al., 1999), as well as increased susceptibility to PD (Lu et al., 2014; Aslam et al., 2017).

**GSTM1** encodes a human glutathione s-transferase. It functions in the detoxification of electrophilic compounds including drugs and environmental toxins. Its association with PD is still up for debate (Wang et al., 2016), some suggested that it linked to PD *via* pesticide exposures (Pinhel et al., 2013). GSTM1 was linked to melanoma (Kanetsky et al., 2001), and the protective effect of coffee consumption was reported to be particularly high for subjects with GSTM1 null polymorphisms (Fortes et al., 2013).

**VDR** encodes the vitamin D receptor, a member of the nuclear receptor family of transcription factors, which binds to the active form of vitamin D. VDR expression was decreased in melanoma (Brożyna et al., 2014), and VDR variants were associated with a higher risk of PD (Butler et al., 2011; Meamar et al., 2017). Its role in PD and melanoma is likely linked to vitamin D deficiency.

**GRIN2A** encodes the regulatory GluN2A subunit of the glutamate-gated N-methyl-d-aspartate receptor (NMDAR). NMDA receptors are a class of ionotropic glutamate receptors found in neurons. Variants of GRIN2A were associated with the protective effect of coffee against PD (Hamza et al., 2011; Yamada-Fowler et al., 2014), and mutations of GRIN2A in melanoma correlate with decreased survival (D’Mello et al., 2014).

The genes discussed above have varied strengths of evidence for their involvement in PD and melanoma, from multidisciplinary studies. To understand their pleiotropic roles in both conditions, it is important to further investigate their biological functions, interactions, and involvements in molecular pathways.

## The Pigmentation Pathway

Dopaminergic neurons and melanocytes share common pathways or cascades that control the final fate of the cells, including cell cycle, DNA repair, oxidative stress, and immune responses. They are both pigmented, and L-dopa is an intermediate product for the synthesis of both dopamine in dopaminergic neurons and melanin in melanocytes.

The associations between red hair color, redhead-related MC1R variants, and risk for both melanoma and PD support a possible role of systemic pigmentation in the PD-melanoma link (Gao et al., 2009b; Edwards et al., 2011; Rumpf et al., 2015; Chen et al., 2017b). In addition, light skin/hair pigmentation was related to abnormal SN echogenicity (Rumpf et al., 2015), and the TYR melanogenesis pathway was identified in the GWAS database as the top significant pathway for PD (Edwards et al., 2011). Furthermore, vitamin D regulates cutaneous pigmentation by enhancing melanogenesis (Pavlovitch et al., 1982; Watabe et al., 2002). PD and melanoma both have vitamin D insufficiency (Sato et al., 1997; Evatt et al., 2008; Field and Newton-Bishop, 2011; Field et al., 2013), and a lower expression level of VDR was found in melanoma (Slominski et al., 2017). These findings suggest that systemic melanogenesis may be compromised in both conditions.

In addition to eumelanin and pheomelanin in the periphery, there is the third melanin—neuromelanin in the human brain. Neuromelanin may be synthesized from dopamine precursor L-dopa, or it could derive from non-enzymatic oxidation in mitochondria from excess cytosolic dopamine accumulated by synaptic vesicles (Zecca et al., 2001). Evidence supports both the protective and cytotoxic effects of neuromelanin (Enochs et al., 1994). PD patients have significantly less neuromelanin in the brain (Zecca et al., 2002). It is unclear whether the decreased level of neuromelanin is a result of the loss of neurons, or the death of neurons is triggered by loss of neuromelanin as its protective function may be compromised. A new study showed the age-dependent production of neuromelanin in rat SN overexpressing TYR. Neuromelanin accumulation was associated with Lewy body-like inclusions and nigrostriatal neurodegeneration (Carballo-Carbajal et al., 2019). The findings that neuromelanin structurally consisted of a pheomelanin core and a eumelanin surface led to a hypothesis that thinning eumelanin surface and exposing pheomelanin core may be responsible for the selective vulnerability of pigmented dopaminergic neurons in PD (Bush et al., 2006; Ito, 2006). Excessive pheomelanin in the periphery (e.g., in red-haired individuals) plays a causal role in melanomagenesis (Roider and Fisher, 2016). It is unknown how neuromelanin might be related to skin melanin or whether MC1R has any role in neuromelanin synthesis. Albino humans with TYR were reported to have pigmented SN (Foley and Baxter, 1958). Further studies should aim to elucidate the exact role of the periphery pigmentation and the central nervous system (CNS) pigmentation in the PD-melanoma association.

A related question is whether L-dopa is involved in high melanoma occurrence in PD. Although bidirectional, the risk ratio for PD preceding melanoma has overall consistently been higher than that for melanoma preceding PD (Liu et al., 2011; Huang et al., 2015), indicating that there might be additional factors contributing to the former. L-dopa as the mainstay therapy for PD and common rate-limiting intermediate products for the biosynthesis of both dopamine in dopaminergic neurons and melanin in melanocytes has long been proposed to be such a contributing factor. In fact, the very first reported melanoma cases in PD were from patients taking levodopa (Skibba et al., 1972). However, subsequent findings that rates of melanoma increased among early PD patients not yet taking L-dopa argue against levodopa’s involvement in this link (Zanetti et al., 2006; Vermeij et al., 2009). This issue has not been fully settled as there have been so far only a few observational studies (Fiala et al., 2003; Constantinescu et al., 2007; Olsen et al., 2007). Melanoma history is still listed as a contraindication to L-dopa use. With the identification of the possible genetic risk factors for both PD and melanoma, it may be necessary to reevaluate L-dopa’s role in melanoma development in PD, especially in populations at risk.

## The Immune System

The immune systems are often weakened in cancers including melanoma (Rangwala and Tsai, 2011). In fact, melanoma is considered the immunogenic tumor, and cell proliferation, as well as apoptosis, are associated with immune system dysfunctions during melanomagenesis (Passarelli et al., 2017). Further, immunotherapy is in use to treat advanced melanoma (Passarelli et al., 2017). Once considered immune-privileged, the CNS, in fact, possesses abilities to elicit immune responses. Neuroinflammation plays a role in the pathogenesis as well as disease progression of PD (Tansey and Goldberg, 2010; Vivekanantham et al., 2015; Gelders et al., 2018). Post-mortem studies found elevated levels of pro-inflammatory factors in the brains of PD patients. Glial cell activation was detected in the brains of PD patients or the animal models of PD. As the disease progresses, α-Syn and other molecules released from the degenerating dopamine neurons may further activate microglia, which may result in more neuron death and thus forming a vicious cycle of neurodegeneration (Wang et al., 2015). Emerging evidence supports the involvement of peripheral inflammation and the peripheral immune system in PD pathogenesis and progression (Garretti et al., 2019; Kustrimovic et al., 2019). Constipation is often a symptom of PD, and PD patients have altered gut microbiome, which may increase inflammation (Garretti et al., 2019). T cells from PD patients respond to α-Syn to a greater degree when compared to the control subjects (Sulzer et al., 2017). A study using human induced pluripotent stem cell (iPSC)-based PD model showed increased neuronal death induced by upregulation of IL-17 receptor (IL-17R) and NF-κB activation, and the neuronal death could be rescued by blockage of IL-17 or IL-17R or the use of an IL-17 antibody (Sommer et al., 2018). More studies are needed to explore immune dysregulation, particularly the systemic immune system and its interactions with the central immune system in the PD-melanoma link.

## Conclusion and Future Perspectives

PD patients have a significantly higher risk of developing melanoma, and vice versa. The association is well-documented by epidemiological studies and supported by clinical and biological studies. PD and melanoma share several risk factors, inverse risk factors, as well as some common clinical features. Genetic studies have identified genes that may be associated with both conditions. Studies using cellular and animal models further provided evidence for a possibly critical role of the pigmentation pathway in this association. As complex as the two pathologies separately, their association is likely a result of complex interactions between environmental and genetic factors. The immune dysregulation may represent one of the convergent mechanisms leading to neurodegeneration in dopaminergic neurons and tumorigenesis in melanocytes. Future studies using detailed systems analysis focusing on the multi-level interactions between PD and melanoma may lead to a more comprehensive understanding of the mechanisms underly the PD-melanoma link, thus shed fresh insight on both pathologies and help develop treatment strategies.

## Author Contributions

QY, YW, NA-K, and XC wrote the article.

## Conflict of Interest

The authors declare that the research was conducted in the absence of any commercial or financial relationships that could be construed as a potential conflict of interest.

## References

[B1] Agalliu I. Ortega R. A. Luciano M. S. Mirelman A. Pont-Sunyer C. Brockmann K.. (2019). Cancer outcomes among Parkinson’s disease patients with leucine rich repeat kinase 2 mutations, idiopathic Parkinson’s disease patients, and nonaffected controls. Mov. Disord. 34, 1392–1398. 10.1002/mds.27807 31348549 PMC6754269

[B2] Agalliu I. San Luciano M. Mirelman A. Giladi N. Waro B. Aasly J.. (2015). Higher frequency of certain cancers in LRRK2 G2019S mutation carriers with Parkinson disease: a pooled analysis. JAMA Neurol. 72, 58–65. 10.1001/jamaneurol.2014.1973 25401981 PMC4366130

[B3] Aguissa-Touré A.-H. Li G. (2012). Genetic alterations of PTEN in human melanoma. Cell. Mol. Life Sci. 69, 1475–1491. 10.1007/s00018-011-0878-0 22076652 PMC11114653

[B4] Alves da Costa C. Checler F. (2011). Apoptosis in Parkinson’s disease: is p53 the missing link between genetic and sporadic Parkinsonism? Cell. Signal. 23, 963–968. 10.1016/j.cellsig.2010.10.020 20969953

[B5] Anwarullah Aslam M. Badshah M. Abbasi R. Sultan A. Khan K.. (2017). Further evidence for the association of CYP2D6*4 gene polymorphism with Parkinson’s disease: a case control study. Genes Environ. 39:18. 10.1186/s41021-017-0078-8 28680508 PMC5493842

[B6] Arena G. Valente E. M. (2017). PINK1 in the limelight: multiple functions of an eclectic protein in human health and disease. J. Pathol. 241, 251–263. 10.1002/path.4815 27701735

[B7] Ascherio A. Schwarzschild M. A. (2016). The epidemiology of Parkinson’s disease: risk factors and prevention. Lancet Neurol. 15, 1257–1272. 10.1016/S1474-4422(16)30230-7 27751556

[B8] Bajaj A. Driver J. A. Schernhammer E. S. (2010). Parkinson’s disease and cancer risk: a systematic review and meta-analysis. Cancer Causes Control 21, 697–707. 10.1007/s10552-009-9497-6 20054708

[B9] Benedetti M. D. Bower J. H. Maraganore D. M. McDonnell S. K. Peterson B. J. Ahlskog J. E.. (2000). Smoking, alcohol and coffee consumption preceding Parkinson’s disease: a case-control study. Neurology 55, 1350–1358. 10.1212/wnl.55.9.1350 11087780

[B10] Bose A. Petsko G. A. Eliezer D. (2018). Parkinson’s disease and melanoma: co-occurrence and mechanisms. J. Parkinsons Dis. 8, 385–398. 10.3233/JPD-171263 29991141 PMC6130416

[B11] Brożyna A. A. Jóźwicki W. Slominski A. T. (2014). Decreased VDR expression in cutaneous melanomas as marker of tumor progression: new data and analyses. Anticancer Res. 34, 2735–2743. 24922634 PMC4273563

[B12] Brown K. Yang P. Salvador D. Kulikauskas R. Ruohola-Baker H. Robitaille A. M.. (2017). WNT/β-catenin signaling regulates mitochondrial activity to alter the oncogenic potential of melanoma in a PTEN-dependent manner. Oncogene 36, 3119–3136. 10.1038/onc.2016.450 28092677 PMC5467017

[B13] Bush W. D. Garguilo J. Zucca F. A. Albertini A. Zecca L. Edwards G. S.. (2006). The surface oxidation potential of human neuromelanin reveals a spherical architecture with a pheomelanin core and a eumelanin surface. Proc. Natl. Acad. Sci. U S A 103, 14785–14789. 10.1073/pnas.0604010103 17001010 PMC1595429

[B14] Butler M. W. Burt A. Edwards T. L. Zuchner S. Scott W. K. Martin E. R.. (2011). Vitamin D receptor gene as a candidate gene for Parkinson disease. Ann. Hum. Genet. 75, 201–210. 10.1111/j.1469-1809.2010.00631.x 21309754 PMC3077063

[B15] Caini S. Masala G. Saieva C. Kvaskoff M. Savoye I. Sacerdote C.. (2017). Coffee, tea and melanoma risk: findings from the European Prospective Investigation into Cancer and Nutrition. Int. J. Cancer 140, 2246–2255. 10.1002/ijc.30659 28218395 PMC6198927

[B16] Carballo-Carbajal I. Laguna A. Romero-Giménez J. Cuadros T. Bové J. Martinez-Vicente M.. (2019). Brain tyrosinase overexpression implicates age-dependent neuromelanin production in Parkinson’s disease pathogenesis. Nat. Commun. 10:973. 10.1038/s41467-019-08858-y 30846695 PMC6405777

[B17] Cartier A. E. Ubhi K. Spencer B. Vazquez-Roque R. A. Kosberg K. A. Fourgeaud L.. (2012). Differential effects of UCHL1 modulation on α-synuclein in PD-like models of α-synucleinopathy. PLoS One 7:e34713. 10.1371/journal.pone.0034713 22514658 PMC3326048

[B18] Cecconi D. Carbonare L. D. Mori A. Cheri S. Deiana M. Brandi J.. (2017). An integrated approach identifies new oncotargets in melanoma. Oncotarget 9, 11489–11502. 10.18632/oncotarget.23727 29545914 PMC5837771

[B21] Chen X. Chen H. Cai W. Maguire M. Ya B. Zuo F.. (2017a). The melanoma-linked “redhead” MC1R influences dopaminergic neuron survival. Ann. Neurol. 81, 395–406. 10.1002/ana.24852 28019657 PMC6085083

[B22] Chen X. Feng D. Schwarzschild M. A. Gao X. (2017b). Red hair, *MC1R* variants, and risk for Parkinson’s disease—a meta-analysis. Ann. Clin. Transl. Neurol. 4, 212–216. 10.1002/acn3.381 28275654 PMC5338132

[B20] Chen L.-L. Tian J. J. Su L. Jing Y. Zhang S. C. Zhang H. X.. (2015). DJ-1: a promising marker in metastatic uveal melanoma. J. Cancer Res. Clin. Oncol. 141, 315–321. 10.1007/s00432-014-1804-2 25129821 PMC11823958

[B19] Chen J.-C. Tsai T.-Y. Li C.-Y. Hwang J.-H. (2015). Obstructive sleep apnea and risk of Parkinson’s disease: a population-based cohort study. J. Sleep Res. 24, 432–437. 10.1111/jsr.12289 25810019

[B23] Chou P.-S. Lai C.-L. Chou Y.-H. Chang W.-P. (2017). Sleep apnea and the subsequent risk of Parkinson’s disease: a 3-year nationwide population-based study. Neuropsychiatr. Dis. Treat. 13, 959–965. 10.2147/NDT.S134311 28408829 PMC5384714

[B24] Constantinescu R. Romer M. Kieburtz K. DATATOP Investigators of the Parkinson Study Group. (2007). Malignant melanoma in early Parkinson’s disease: the DATATOP trial. Mov. Disord. 22, 720–722. 10.1002/mds.21273 17373726

[B25] Costa J. Lunet N. Santos C. Santos J. Vaz-Carneiro A. (2010). Caffeine exposure and the risk of Parkinson’s disease: a systematic review and meta-analysis of observational studiess. J. Alzheimers Dis. 20, S221–S238. 10.3233/JAD-2010-091525 20182023

[B26] Cui X. Liew Z. Hansen J. Lee P. C. Arah O. A. Ritz B. (2019). Cancers preceding Parkinson’s disease after adjustment for bias in a danish population-based case-control study. Neuroepidemiology 52, 136–143. 10.1159/000494292 30661072 PMC6482096

[B27] D’Mello S. A. N. Flanagan J. U. Green T. N. Leung E. Y. Askarian-Amiri M. E. Joseph W. R.. (2014). Evidence that GRIN2A mutations in melanoma correlate with decreased survival. Front. Oncol. 3:333. 10.3389/fonc.2013.00333 24455489 PMC3888952

[B28] Dahodwala N. Siderowf A. Xie M. Noll E. Stern M. Mandell D. S. (2009). Racial differences in the diagnosis of Parkinson’s disease. Mov. Disord. 24, 1200–1205. 10.1002/mds.22557 19412929 PMC2858583

[B29] Dennis L. K. Lynch C. F. Sandler D. P. Alavanja M. C. R. (2010). Pesticide use and cutaneous melanoma in pesticide applicators in the agricultural heath study. Environ. Health Perspect. 118, 812–817. 10.1289/ehp.0901518 20164001 PMC2898858

[B30] Desideri E. Martins L. M. (2012). Mitochondrial stress signalling: HTRA2 and Parkinson’s disease. Int. J. Cell Biol. 2012:607929. 10.1155/2012/607929 22675361 PMC3362845

[B31] Dusingize J. C. Olsen C. M. Pandeya N. Thompson B. S. Webb P. M. Green A. C.. (2018). Smoking and cutaneous melanoma: findings from the QSkin sun and health cohort study. Cancer Epidemiol. Biomarkers Prev. 27, 874–881. 10.1158/1055-9965.epi-17-1056 29789324

[B32] Edwards Y. J. K. Beecham G. W. Scott W. K. Khuri S. Bademci G. Tekin D.. (2011). Identifying consensus disease pathways in Parkinson’s disease using an integrative systems biology approach. PLoS One 6:e16917. 10.1371/journal.pone.0016917 21364952 PMC3043094

[B33] Enochs W. S. Sarna T. Zecca L. Riley P. A. Swartz H. M. (1994). The roles of neuromelanin, binding of metal ions, and oxidative cytotoxicity in the pathogenesis of Parkinson’s disease: a hypothesis. J. Neural Transm. Park. Dis. Dement. Sect. 7, 83–100. 10.1007/bf02260963 7710667

[B34] Evatt M. L. Delong M. R. Khazai N. Rosen A. Triche S. Tangpricha V. (2008). Prevalence of vitamin D insufficiency in patients with parkinson disease and Alzheimer disease. Arch. Neurol. 65, 1348–1352. 10.1001/archneur.65.10.1348 18852350 PMC2746037

[B35] Fiala K. H. Whetteckey J. Manyam B. V. (2003). Malignant melanoma and levodopa in Parkinson’s disease: causality or coincidence? Phys. Med. Biol. 9, 321–327. 10.1016/s1353-8020(03)00040-3 12853231

[B37] Field S. Davies J. Bishop D. T. Newton-Bishop J. A. (2013). Vitamin D and melanoma. Dermatoendocrinol. 5, 121–129. 10.4161/derm.25244 24494045 PMC3897580

[B36] Field S. Newton-Bishop J. A. (2011). Melanoma and vitamin D. Mol. Oncol. 5, 197–214. 10.1016/j.molonc.2011.01.007 21371954 PMC5528277

[B38] Fois A. F. Wotton C. J. Yeates D. Turner M. R. Goldacre M. J. (2010). Cancer in patients with motor neuron disease, multiple sclerosis and Parkinson’s disease: record linkage studies. J. Neurol. Neurosurg. Psychiatry 81, 215–221. 10.1136/jnnp.2009 19726405

[B39] Foley J. M. Baxter D. (1958). On the nature of pigment granules in the cells of the locus coeruleus and substantia nigra. J. Neuropathol. Exp. Neurol. 17, 586–598. 10.1097/00005072-195810000-00005 13588387

[B40] Fortes C. Mastroeni S. Boffetta P. Antonelli G. Pilla M. A. Bottà G.. (2013). The protective effect of coffee consumption on cutaneous melanoma risk and the role of GSTM1 and GSTT1 polymorphisms. Cancer Causes Control 24, 1779–1787. 10.1007/s10552-013-0255-4 23860951

[B41] Gao X. Simon K. C. Han J. Schwarzschild M. A. Ascherio A. (2009a). Family history of melanoma and Parkinson disease risk. Neurology 73, 1286–1291. 10.1212/WNL.0b013e3181bd13a1 19841380 PMC2764417

[B42] Gao X. Simon K. C. Han J. Schwarzschild M. A. Ascherio A. (2009b). Genetic determinants of hair color and Parkinson’s disease risk. Ann. Neurol. 65, 76–82. 10.1002/ana.21535 19194882 PMC2639631

[B43] Garretti F. Agalliu D. Lindestam Arlehamn C. S. Sette A. Sulzer D. (2019). Autoimmunity in Parkinson’s disease: the role of α-synuclein-specific T cells. Front. Immunol. 10:303. 10.3389/fimmu.2019.00303 30858851 PMC6397885

[B44] Gelders G. Baekelandt V. Van der Perren A. (2018). Linking neuroinflammation and neurodegeneration in Parkinson’s disease. J. Immunol. Res. 2018:4784268. 10.1155/2018/4784268 29850629 PMC5926497

[B45] Goldman S. M. (2014). Environmental toxins and Parkinson’s disease. Annu. Rev. Pharmacol. Toxicol. 54, 141–164. 10.1146/annurev-pharmtox-011613-135937 24050700

[B46] Gozal D. Ham S. A. Mokhlesi B. (2016). Sleep apnea and cancer: analysis of a nationwide population sample. Sleep 39, 1493–1500. 10.5665/sleep.6004 27166241 PMC4945307

[B47] Guo H. Carlson J. A. Slominski A. (2012). Role of TRPM in melanocytes and melanoma. Exp. Dermatol. 21, 650–654. 10.1111/j.1600-0625.2012.01565.x 22897572 PMC3422761

[B48] Hamza T. H. Chen H. Hill-Burns E. M. Rhodes S. L. Montimurro J. Kay D. M.. (2011). Genome-wide gene-environment study identifies glutamate receptor gene GRIN2A as a Parkinson’s disease modifier gene *via* interaction with coffee. PLoS Genet. 7:e1002237. 10.1371/journal.pgen.1002237 21876681 PMC3158052

[B49] Hernán M. A. Takkouche B. Caamaño-Isorna F. Gestal-Otero J. J. (2002). A meta-analysis of coffee drinking, cigarette smoking, and the risk of Parkinson’s disease. Ann. Neurol. 52, 276–284. 10.1002/ana.10277 12205639

[B50] Hernán M. A. Zhang S. M. Rueda-deCastro A. M. Colditz G. A. Speizer F. E. Ascherio A. (2001). Cigarette smoking and the incidence of Parkinson’s disease in two prospective studies. Ann. Neurol. 50, 780–786. 10.1002/ana.10028 11761476

[B51] Hu G. Bidel S. Jousilahti P. Antikainen R. Tuomilehto J. (2007). Coffee and tea consumption and the risk of Parkinson’s disease. Mov. Disord. 22, 2242–2248. 10.1002/mds.21706 17712848

[B52] Hu H.-H. Kannengiesser C. Lesage S. André J. Mourah S. Michel L.. (2016). PARKIN inactivation links Parkinson’s disease to melanoma. J. Natl. Cancer Inst. 108:djv340. 10.1093/jnci/djv340 26683220

[B53] Huang P. Yang X.-D. Chen S.-D. Xiao Q. (2015). The association between Parkinson’s disease and melanoma: a systematic review and meta-analysis. Transl. Neurodegener. 4:21. 10.1186/s40035-015-0044-y 26535116 PMC4631109

[B54] Inzelberg R. Cohen O. S. Aharon-Peretz J. Schlesinger I. Gershoni-Baruch R. Djaldetti R.. (2012). The LRRK2 G2019S mutation is associated with Parkinson disease and concomitant non-skin cancers. Neurology 78, 781–786. 10.1212/wnl.0b013e318249f673 22323743

[B55] Inzelberg R. Flash S. Friedman E. Azizi E. (2016a). Cutaneous malignant melanoma and Parkinson disease: common pathways? Ann. Neurol. 80, 811–820. 10.1002/ana.24802 27761938

[B56] Inzelberg R. Samuels Y. Azizi E. Qutob N. Inzelberg L. Domany E.. (2016b). Parkinson disease (PARK) genes are somatically mutated in cutaneous melanoma. Neurol. Genet. 2:e70. 10.1212/nxg.0000000000000070 27123489 PMC4832432

[B57] Ito S. (2006). Encapsulation of a reactive core in neuromelanin. Proc. Natl. Acad. Sci. U S A 103, 14647–14648. 10.1073/pnas.0606879103 17005730 PMC1595404

[B58] Jones M. S. Jones P. C. Stern S. L. Elashoff D. Hoon D. S. B. Thompson J.. (2017). The impact of smoking on sentinel node metastasis of primary cutaneous melanoma. Ann. Surg. Oncol. 24, 2089–2094. 10.1245/s10434-017-5775-9 28224364 PMC5553293

[B59] Kanetsky P. A. Holmes R. Walker A. Najarian D. Swoyer J. Guerry D.. (2001). Interaction of glutathione S-transferase M1 and T1 genotypes and malignant melanoma. Cancer Epidemiol. Biomarkers Prev. 10, 509–513. 11352862

[B60] Kessides M. C. Wheless L. Hoffman-Bolton J. Clipp S. Alani R. M. Alberg A. J. (2011). Cigarette smoking and malignant melanoma: a case-control study. J. Am. Acad. Dermatol. 64, 84–90. 10.1016/j.jaad.2010.01.041 20334951 PMC2924442

[B61] Kun-Rodrigues C. Ganos C. Guerreiro R. Schneider S. A. Schulte C. Lesage S.. (2015). A systematic screening to identify *de novo* mutations causing sporadic early-onset Parkinson’s disease. Hum. Mol. Genet. 24, 6711–6720. 10.1093/hmg/ddv376 26362251 PMC4634375

[B62] Kustrimovic N. Marino F. Cosentino M. (2019). Peripheral immunity, immunoaging and neuroinflammation in Parkinson’s disease. Curr. Med. Chem. 26, 3719–3753. 10.2174/0929867325666181009161048 30306855

[B63] Law M. H. MacGregor S. Hayward N. K. (2012). Melanoma genetics: recent findings take us beyond well-traveled pathways. J. Invest. Dermatol. 132, 1763–1774. 10.1038/jid.2012.75 22475760

[B64] Li X. Li W. Liu G. Shen X. Tang Y. (2015). Association between cigarette smoking and Parkinson’s disease: a meta-analysis. Arch. Gerontol. Geriatr. 61, 510–516. 10.1016/j.archger.2015.08.004 26272284

[B65] Lin G. Lee P. T. Chen K. Mao D. Tan K. L. Zuo Z.. (2018). Phospholipase PLA2G6, a Parkinsonism-associated gene, affects Vps26 and Vps35, retromer function, and ceramide levels, similar to α-synuclein gain. Cell Metab. 28, 605.e6–618.e6. 10.1016/j.cmet.2018.05.019 29909971

[B66] Liu R. Gao X. Lu Y. Chen H. (2011). Meta-analysis of the relationship between Parkinson disease and melanoma. Neurology 76, 2002–2009. 10.1212/wnl.0b013e31821e554e 21646627 PMC3116643

[B67] Loftfield E. Freedman N. D. Graubard B. I. Hollenbeck A. R. Shebl F. M. Mayne S. T.. (2015). Coffee drinking and cutaneous melanoma risk in the NIH-AARP diet and health study. J. Natl. Cancer Inst. 107:dju421. 10.1093/jnci/dju421 25604135 PMC4311176

[B68] Lu Y. Peng Q. Zeng Z. Wang J. Deng Y. Xie L.. (2014). CYP2D6 phenotypes and Parkinson’s disease risk: a meta-analysis. J. Neurol. Sci. 336, 161–168. 10.1016/j.jns.2013.10.030 24211060

[B69] Lukic M. Jareid M. Weiderpass E. Braaten T. (2016). Coffee consumption and the risk of malignant melanoma in the Norwegian Women and Cancer (NOWAC) study. BMC Cancer 16:562. 10.1186/s12885-016-2586-5 27473841 PMC4966737

[B70] Maraganore D. M. Lesnick T. G. Elbaz A. Chartier-Harlin M. C. Gasser T. Krüger R.. (2004). UCHL1 is a Parkinson’s disease susceptibility gene. Ann. Neurol. 55, 512–521. 10.1002/ana.20017 15048890

[B71] Martínez-García M.-Á. Martorell-Calatayud A. Nagore E. Valero I. Selma M. J. Chiner E.. (2014). Association between sleep disordered breathing and aggressiveness markers of malignant cutaneous melanoma. Eur. Respir. J. 43, 1661–1668. 10.1183/09031936.00115413 24659545

[B72] Martinez-Garcia M. A. Campos-Rodriguez F. Nagore E. Martorell A. Rodriguez-Peralto J. L. Riveiro-Falkenbach E.. (2018). Sleep-disordered breathing is independently associated with increased aggressiveness of cutaneous melanoma: a multicenter observational study in 443 patients. Chest 154, 1348–1358. 10.1016/j.chest.2018.07.015 30059679

[B73] Matsuo Y. Kamitani T. (2010). Parkinson’s disease-related protein, α-synuclein, in malignant melanoma. PLoS One 5:e10481. 10.1371/journal.pone.0010481 20463956 PMC2864738

[B74] Meamar R. Javadirad S. M. Chitsaz N. Ghahfarokhi M. A. Kazemi M. Ostadsharif M. (2017). Vitamin D receptor gene variants in Parkinson’s disease patients. Egypt. J. Med. Hum. Genet. 18, 225–230. 10.1016/j.ejmhg.2016.08.004

[B76] Menza M. Dobkin R. D. Marin H. Bienfait K. (2010). Sleep disturbances in Parkinson’s disease. Mov. Disord. 25, S117–S122. 10.1002/mds.22788 20187236 PMC2840057

[B77] Moreno-Arrones O. M. Zegeer J. Gerbo M. Manrique-Silva E. Requena C. Traves V.. (2019). Decreased vitamin D serum levels at melanoma diagnosis are associated with tumor ulceration and high tumor mitotic rate. Melanoma Res. 29, 664–667. 10.1097/cmr.0000000000000638 31469708

[B78] Murthy M. N. Blauwendraat C. UKBEC Guelfi S. IPDGC Hardy J.. (2017). Increased brain expression of GPNMB is associated with genome wide significant risk for Parkinson’s disease on chromosome 7p15.3. Neurogenetics 18, 121–133. 10.1007/s10048-017-0514-8 28391543 PMC5522530

[B79] Nandipati S. Litvan I. (2016). Environmental exposures and Parkinson’s disease. Int. J. Environ. Res. Public Health 13:E881. 10.3390/ijerph13090881 27598189 PMC5036714

[B75] National Cancer Institute (2019). SEER Cancer Stat Facts: Melanoma Skin Cancer Statistics. Available online at: https://www.cancer.org/cancer/melanoma-skin-cancer/about/key-statistics.html. Accessed June 10, 2019.

[B80] O’Reilly E. J. Chen H. Gardener H. Gao X. Schwarzschild M. A. Ascherio A. (2009). Smoking and Parkinson’s disease: using parental smoking as a proxy to explore causality. Am. J. Epidemiol. 169, 678–682. 10.1093/aje/kwn388 19131566 PMC2727210

[B81] O’Reilly E. J. McCullough M. L. Chao A. Henley S. J. Calle E. E. Thun M. J.. (2005). Smokeless tobacco use and the risk of Parkinson’s disease mortality. Mov. Disord. 20, 1383–1384. 10.1002/mds.20587 16007624

[B82] Olsen J. H. Tangerud K. Wermuth L. Frederiksen K. Friis S. (2007). Treatment with levodopa and risk for malignant melanoma. Mov. Disord. 22, 1252–1257. 10.1002/mds.21397 17534943

[B83] Pan T. Zhu J. Hwu W.-J. Jankovic J. (2012). The role of α-synuclein in melanin synthesis in melanoma and dopaminergic neuronal cells. PLoS One 7:e45183. 10.1371/journal.pone.0045183 23028833 PMC3446957

[B84] Passarelli A. Mannavola F. Stucci L. S. Tucci M. Silvestris F. (2017). Immune system and melanoma biology: a balance between immunosurveillance and immune escape. Oncotarget 8, 106132–106142. 10.18632/oncotarget.22190 29285320 PMC5739707

[B85] Pavlovitch J. H. Rizk M. Balsan S. (1982). Vitamin D nutrition increases skin tyrosinase response to exposure to ultraviolet radiation. Mol. Cell. Endocrinol. 25, 295–302. 10.1016/0303-7207(82)90085-5 6175546

[B86] Peterson A. L. Murchison C. Zabetian C. Leverenz J. B. Watson G. S. Montine T.. (2013). Memory, mood, and vitamin D in persons with Parkinson’s disease. J. Parkinsons Dis. 3, 547–555. 10.3233/JPD-130206 24081441 PMC3966187

[B87] Pinhel M. A. S. Sado C. L. Longo Gdos S. Gregório M. L. Amorim G. S. Florim G. M.. (2013). Nullity of GSTT1/GSTM1 related to pesticides is associated with Parkinson’s disease. Arq. Neuropsiquiatr. 71, 527–532. 10.1590/0004-282x20130076 23982010

[B88] Plun-Favreau H. Klupsch K. Moisoi N. Gandhi S. Kjaer S. Frith D.. (2007). The mitochondrial protease HtrA2 is regulated by Parkinson’s disease-associated kinase PINK1. Nat. Cell Biol. 9, 1243–1252. 10.1038/ncb1644 17906618

[B89] Rangwala S. Tsai K. Y. (2011). Roles of the immune system in skin cancer. Br. J. Dermatol. 165, 953–965. 10.1111/j.1365-2133.2011.10507.x 21729024 PMC3197980

[B90] Rastrelli M. Tropea S. Rossi C. R. Alaibac M. (2014). Melanoma: epidemiology, risk factors, pathogenesis, diagnosis and classification. In Vivo 28, 1005–1011. 25398793

[B91] Ritz B. Ascherio A. Checkoway H. Marder K. S. Nelson L. M. Rocca W. A.. (2007). Pooled analysis of tobacco use and risk of Parkinson disease. Arch. Neurol. 64, 990–997. 10.1001/archneur.64.7.990 17620489

[B92] Rodríguez-Leyva I. Calderón-Garcidueñas A. L. Jiménez-Capdeville M. E. Rentería-Palomo A. A. Hernandez-Rodriguez H. G. Valdés-Rodríguez R.. (2014). α-synuclein inclusions in the skin of Parkinson’s disease and parkinsonism. Ann. Clin. Transl. Neurol. 1, 471–478. 10.1002/acn3.78 25356418 PMC4184776

[B93] Rodriguez-Leyva I. Chi-Ahumada E. Mejía M. Castanedo-Cazares J. P. Eng W. Saikaly S. K.. (2017). The presence of α-synuclein in skin from melanoma and patients with Parkinson’s disease. Mov. Disord. Clin. Pract. 4, 724–732. 10.1002/mdc3.12494 30363411 PMC6174388

[B94] Roider E. M. Fisher D. E. (2016). Red hair, light skin, and UV-independent risk for melanoma development in humans. JAMA Dermatol. 152, 751–753. 10.1001/jamadermatol.2016.0524 27050924 PMC5241673

[B95] Rumpf J.-J. Schirmer M. Fricke C. Weise D. Wagner J. A. Simon J.. (2015). Light pigmentation phenotype is correlated with increased substantia nigra echogenicity. Mov. Disord. 30, 1848–1852. 10.1002/mds.26427 26395561

[B96] Rumpf J.-J. Weise D. Fricke C. Wetzig T. Simon J. C. Classen J. (2013). Sonographic abnormality of the substantia nigra in melanoma patients. Mov. Disord. 28, 219–223. 10.1002/mds.25233 23114984

[B97] Sato Y. Kikuyama M. Oizumi K. (1997). High prevalence of vitamin D deficiency and reduced bone mass in Parkinson’s disease. Neurology 49, 1273–1278. 10.1212/wnl.49.5.1273 9371907

[B98] Searles Nielsen S. Gallagher L. G. Lundin J. I. Longstreth W. T. Jr. Smith-Weller T. Franklin G. M.. (2012). Environmental tobacco smoke and Parkinson’s disease. Mov. Disord. 27, 293–296. 10.1002/mds.24012 22095755 PMC3289937

[B99] Seo E. Y. Jin S. P. Sohn K. C. Park C. H. Lee D. H. Chung J. H. (2017). UCHL1 regulates melanogenesis through controlling MITF Stability in human melanocytes. J. Invest. Dermatol. 137, 1757–1765. 10.1016/j.jid.2017.03.024 28392346

[B100] Skibba J. L. Pinckley J. Gilbert E. F. Johnson R. O. (1972). Multiple primary melanoma following administration of levodopa. Arch. Pathol. 93, 556–561. 5025039

[B101] Sleeman I. Aspray T. Lawson R. Coleman S. Duncan G. Khoo T. K.. (2017). The role of vitamin D in disease progression in early Parkinson’s disease. J. Parkinsons Dis. 7, 669–675. 10.3233/JPD-171122 28984616 PMC5676984

[B102] Slominski A. T. Brożyna A. A. Zmijewski M. A. Jóźwicki W. Jetten A. M. Mason R. S.. (2017). Vitamin D signaling and melanoma: role of vitamin D and its receptors in melanoma progression and management. Lab. Invest. 97, 706–724. 10.1038/labinvest.2017.3 28218743 PMC5446295

[B103] Sommer A. Marxreiter F. Krach F. Fadler T. Grosch J. Maroni M.. (2018). Th17 lymphocytes induce neuronal cell death in a human iPSC-based model of Parkinson’s disease. Cell Stem Cell 23, 123.e6–131.e6. 10.1016/j.stem.2018.06.015 29979986

[B104] Strange R. C. Ellison T. Ichii-Jones F. Bath J. Hoban P. Lear J. T.. (1999). Cytochrome P450 CYP2D6 genotypes: association with hair colour, Breslow thickness and melanocyte stimulating hormone receptor alleles in patients with malignant melanoma. Pharmacogenetics 9, 269–276. 10.1097/00008571-199906000-00001 10471058

[B105] Strauss K. M. Martins L. M. Plun-Favreau H. Marx F. P. Kautzmann S. Berg D.. (2005). Loss of function mutations in the gene encoding Omi/HtrA2 in Parkinson’s disease. Hum. Mol. Genet. 14, 2099–2111. 10.1093/hmg/ddi215 15961413

[B106] Su D. M. Zhang Q. Wang X. He P. Zhu Y. J. Zhao J.. (2009). Two types of human malignant melanoma cell lines revealed by expression patterns of mitochondrial and survival-apoptosis genes: implications for malignant melanoma therapy. Mol. Cancer Ther. 8, 1292–1304. 10.1158/1535-7163.mct-08-1030 19383853 PMC3128982

[B107] Sulzer D. Alcalay R. N. Garretti F. Cote L. Kanter E. Agin-Liebes J.. (2017). T cells from patients with Parkinson’s disease recognize α-synuclein peptides. Nature 546, 656–661. 10.1038/nature22815 28636593 PMC5626019

[B108] Sun Y. Sukumaran P. Schaar A. Singh B. B. (2015). TRPM7 and its role in neurodegenerative diseases. Channels 9, 253–261. 10.1080/19336950.2015.1075675 26218331 PMC4826135

[B109] Tacik P. Curry S. Fujioka S. Strongosky A. Uitti R. J. van Gerpen J. A.. (2016). Cancer in Parkinson’s disease. Parkinsonism Relat. Disord. 31, 28–33. 10.1016/j.parkreldis.2016.06.014 27372241 PMC5048511

[B110] Tansey M. G. Goldberg M. S. (2010). Neuroinflammation in Parkinson’s disease: its role in neuronal death and implications for therapeutic intervention. Neurobiol. Dis. 37, 510–518. 10.1016/j.nbd.2009.11.004 19913097 PMC2823829

[B111] Taya M. Hammes S. R. (2018). Glycoprotein non-metastatic melanoma protein B (GPNMB) and cancer: a novel potential therapeutic target. Steroids 133, 102–107. 10.1016/j.steroids.2017.10.013 29097143 PMC6166407

[B112] Timerman D. McEnery-Stonelake M. Joyce C. J. Nambudiri V. E. Hodi F. S. Claus E. B.. (2016). Vitamin D deficiency is associated with a worse prognosis in metastatic melanoma. Oncotarget 8, 6873–6882. 10.18632/oncotarget.14316 28036288 PMC5351676

[B113] Tomiyama H. Yoshino H. Ogaki K. Li L. Yamashita C. Li Y.. (2011). PLA2G6 variant in Parkinson’s disease. J. Hum. Genet. 56, 401–403. 10.1038/jhg.2011.22 21368765

[B114] Van Den Eeden S. K. Tanner C. M. Bernstein A. L. Fross R. D. Leimpeter A. Bloch D. A.. (2003). Incidence of Parkinson’s disease: variation by age, gender, and race/ethnicity. Am. J. Epidemiol. 157, 1015–1022. 10.1093/aje/kwg068 12777365

[B115] Vasseur S. Afzal S. Tardivel-Lacombe J. Park D. S. Iovanna J. L. Mak T. W. (2009). DJ-1/PARK7 is an important mediator of hypoxia-induced cellular responses. Proc. Natl. Acad. Sci. U S A 106, 1111–1116. 10.1073/pnas.0812745106 19144925 PMC2626605

[B116] Vermeij J.-D. Winogrodzka A. Trip J. Weber W. E. J. (2009). Parkinson’s disease, levodopa-use and the risk of melanoma. Parkinsonism Relat. Disord. 15, 551–553. 10.1016/j.parkreldis.2009.05.002 19501540

[B117] Vivekanantham S. Shah S. Dewji R. Dewji A. Khatri C. Ologunde R. (2015). Neuroinflammation in Parkinson’s disease: role in neurodegeneration and tissue repair. Int. J. Neurosci. 125, 717–725. 10.3109/00207454.2014.982795 25364880

[B118] Wahabi K. Perwez A. Rizvi M. A. (2018). Parkin in Parkinson’s disease and cancer: a double-edged sword. Mol. Neurobiol. 55, 6788–6800. 10.1007/s12035-018-0879-1 29349575

[B119] Walter U. Heilmann E. Voss J. Riedel K. Zhivov A. Schäd S. G.. (2016). Frequency and profile of Parkinson’s disease prodromi in patients with malignant melanoma. J. Neurol. Neurosurg. Psychiatry 87, 302–310. 10.1136/jnnp-2014-310239 25817520

[B121] Wang Q. Liu Y. Zhou J. (2015). Neuroinflammation in Parkinson’s disease and its potential as therapeutic target. Transl. Neurodegener. 4:19. 10.1186/s40035-015-0042-0 26464797 PMC4603346

[B120] Wang D. Zhai J.-X. Liu D.-W. (2016). Glutathione S-transferase M1 polymorphisms and Parkinson’s disease risk: a meta-analysis. Neurol. Res. 38, 144–150. 10.1080/01616412.2015.1126996 27078701

[B122] Ward W. H. Lambreton F. Goel N. Yu J. Q. Farma J. M. (2017). “Chapter 6-Clinical presentation and staging of melanoma,” in Cutaneous Melanoma: Etiology and Therapy, eds Ward W. H. Farma J. M. (Brisbane, AU: Codon Publications), 79–89. 29461773

[B123] Watabe H. Soma Y. Kawa Y. Ito M. Ooka S. Ohsumi K.. (2002). Differentiation of murine melanocyte precursors induced by 1,25-dihydroxyvitamin D3 is associated with the stimulation of endothelin B receptor expression. J. Invest. Dermatol. 119, 583–589. 10.1046/j.1523-1747.2002.00116.x 12230499

[B124] Welinder C. Jönsson G. B. Ingvar C. Lundgren L. Baldetorp B. Olsson H.. (2014). Analysis of α-synuclein in malignant melanoma—development of a SRM quantification assay. PLoS One 9:e110804. 10.1371/journal.pone.0110804 25333933 PMC4204935

[B125] Wooten G. F. Currie L. J. Bovbjerg V. E. Lee J. K. Patrie J. (2004). Are men at greater risk for Parkinson’s disease than women? J. Neurol. Neurosurg. Psychiatry 75, 637–639. 10.1136/jnnp.2003.020982 15026515 PMC1739032

[B126] Wright Willis A. Evanoff B. A. Lian M. Criswell S. R. Racette B. A. (2010). Geographic and ethnic variation in Parkinson disease: a population-based study of US medicare beneficiaries. Neuroepidemiology 34, 143–151. 10.1159/000275491 20090375 PMC2865395

[B127] Wu S. Han J. Song F. Cho E. Gao X. Hunter D. J.. (2015). Caffeine intake, coffee consumption, and risk of cutaneous malignant melanoma. Epidemiology 26, 898–908. 10.1097/ede.0000000000000360 26172864 PMC4600068

[B128] Wulfänger J. Biehl K. Tetzner A. Wild P. Ikenberg K. Meyer S.. (2013). Heterogeneous expression and functional relevance of the ubiquitin carboxyl-terminal hydrolase L1 in melanoma. Int. J. Cancer 133, 2522–2532. 10.1002/ijc.28278 23686552

[B129] Xu S. Chan P. (2015). Interaction between neuromelanin and α-synuclein in Parkinson’s disease. Biomolecules 5, 1122–1142. 10.3390/biom5021122 26057626 PMC4496713

[B130] Yamada-Fowler N. Fredrikson M. Söderkvist P. (2014). Caffeine interaction with glutamate receptor gene GRIN2A: Parkinson’s disease in swedish population. PLoS One 9:e99294. 10.1371/journal.pone.0099294 24915238 PMC4051678

[B131] Yeh N.-C. Tien K.-J. Yang C.-M. Wang J.-J. Weng S.-F. (2016). Increased risk of Parkinson’s disease in patients with obstructive sleep apnea: a population-based, propensity score-matched, longitudinal follow-up study. Medicine 95:e2293. 10.1097/MD.0000000000002293 26765405 PMC4718231

[B132] Zanetti R. Loria D. Rosso S. (2006). Melanoma, Parkinson’s disease and levodopa: causal or spurious link? A review of the literature. Melanoma Res. 16, 201–206. 10.1097/01.cmr.0000215043.61306.d7 16718266

[B133] Zecca L. Fariello R. Riederer P. Sulzer D. Gatti A. Tampellini D. (2002). The absolute concentration of nigral neuromelanin, assayed by a new sensitive method, increases throughout the life and is dramatically decreased in Parkinson’s disease. FEBS Lett. 510, 216–220. 10.1016/s0014-5793(01)03269-0 11801257

[B134] Zecca L. Tampellini D. Gerlach M. Riederer P. Fariello R. G. Sulzer D. (2001). Substantia nigra neuromelanin: structure, synthesis, and molecular behaviour. Mol. Pathol. 54, 414–418. 11724917 PMC1187132

[B136] Zhang P. Liu B. (2019). Association between Parkinson’s disease and risk of cancer: a PRISMA-compliant meta-analysis. ACS Chem. Neurosci. 10, 4430–4439. 10.1021/acschemneuro.9b00498 31584793

[B135] Zhang H.-J. Zhang J. R. Mao C. J. Li K. Wang F. Chen J.. (2019). Relationship between 25-Hydroxyvitamin D, bone density, and Parkinson’s disease symptoms. Acta Neurol. Scand. 140, 274–280. 10.1111/ane.13141 31389003

